# Diagnosis and treatment outcome of mycotic keratitis at a tertiary eye care center in eastern india

**DOI:** 10.1186/1471-2415-11-39

**Published:** 2011-12-22

**Authors:** Bibhudutta Rautaraya, Savitri Sharma, Sarita Kar, Sujata Das, Srikant K Sahu

**Affiliations:** 1 Ocular Microbiology Service, L. V. Prasad Eye Institute, Bhubaneswar-751024, Odisha, India; 2 Cornea and Anterior Segment Service, L. V. Prasad Eye Institute, Bhubaneswar-751024, Odisha, India

**Keywords:** Mycotic, fungal, keratitis, microscopy, culture, treatment outcome

## Abstract

**Background:**

Mycotic keratitis is an important cause of corneal blindness world over including India. Geographical location and climate are known to influence the profile of fungal diseases. While there are several reports on mycotic keratitis from southern India, comprehensive clinico-microbiological reports from eastern India are few. The reported prevalence of mycotic keratitis are 36.7%,36.3%,25.6%,7.3% in southern, western, north- eastern and northern India respectively. This study reports the epidemiological characteristics, microbiological diagnosis and treatment outcome of mycotic keratitis at a tertiary eye care center in eastern India.

**Methods:**

A retrospective review of medical and microbiology records was done for all patients with laboratory proven fungal keratitis.

**Results:**

Between July 2006 and December 2009, 997 patients were clinically diagnosed as microbial keratitis. While no organisms were found in 25.4% (253/997) corneal samples, 23.4% (233/997) were bacterial, 26.4% (264/997) were fungal (45 cases mixed with bacteria), 1.4% (14/997) were *Acanthamoeba *with or without bacteria and 23.4% (233/997) were microsporidial with or without bacteria. Two hundred fifteen of 264 (81.4%, 215/264) samples grew fungus in culture while 49 corneal scrapings were positive for fungal elements only in direct microscopy. Clinical diagnosis of fungal keratitis was made in 186 of 264 (70.5%) cases. The microscopic detection of fungal elements was achieved by 10% potassium hydroxide with 0.1% calcoflour white stain in 94.8%(238/251) cases. *Aspergillus *species (27.9%, 60/215) and *Fusarium *species (23.2%, 50/215) were the major fungal isolates. Concomitant bacterial infection was seen in 45 (17.1%, 45/264) cases of mycotic keratitis. Clinical outcome of healed scar was achieved in 94 (35.6%, 94/264) cases. Fifty two patients (19.7%, 52/264) required therapeutic PK, 9 (3.4%, 9/264) went for evisceration, 18.9% (50/264) received glue application with bandage contact lens (BCL) for impending perforation, 6.1% (16/264) were unchanged and 16.3% (43/264) were lost to follow up. Poor prognosis like PK (40/52, 75.9%, p < 0.001) and BCL (30/50, 60%, p < 0.001) was seen in significantly larger number of patients with late presentation (> 10 days).

**Conclusions:**

The relative prevalence of mycotic keratitis in eastern India is lower than southern, western and north-eastern India but higher than northern India, however, *Aspergillus *and *Fusarium *are the predominant genera associated with fungal keratitis across India. The response to medical treatment is poor in patients with late presentation.

## Background

Corneal blindness is a major public health problem worldwide and infectious keratitis is one of the predominant causes. Certain conditions like trauma to the eyeball and therapy with antibiotics and corticosteroids render the eye susceptible to infection with various fungi especially in tropical parts of the world [1]. A large number of studies from India have reported epidemiological and microbiological profile of fungal keratitis [1-8], however, there are only few that have provided a comprehensive analysis of the clinical and laboratory profile [5,7]. Minor differences in the frequency and spectrum of fungi associated with mycotic keratitis have been reported from southern (36.7%) [2] northern (7.3%) [4] western (36.3%) [6] and north-eastern (25.6%) [7] India. Both the studies from northeastern India have reported high prevalence (38% and 42%) of fungal keratitis in the region [7,8]. Knowledge of these differences coupled with their corresponding epidemiological features, clinical features and treatment outcome is likely to help the ophthalmologists manage this challenging disease in their area. Comprehensive periodic reports from different geographical areas would help record the variations over a period of time and at the same time provide current diagnostic and management strategies with the possible outcome.

This study presents the wide-ranging clinical and microbiological analysis of 264 cases of mycotic keratitis seen over three and half years period at a tertiary eye care centre in eastern India where all patients were investigated and treated with a uniform protocol.

## Methods

A retrospective analysis was performed for all patients seen between July 2006 and December 2009 with laboratory-proven fungal keratitis. This study was approved by institutional review board of L V Prasad Eye Institute (Ethics Ref. No. LEC 11-071). Documentation of all patients included socio-demographic features, duration of symptoms, predisposing factors, slit lamp biomicroscopy findings, associated ocular conditions, other systemic diseases, therapy received prior to presentation, visual acuity at the time of presentation, treatment given, response to treatment during follow up and the clinical outcome. Based on duration of symptoms the patients were divided in to early onset (≤ 10 days) or late onset (> 10 days) disease.

Corneal scrapings were collected and processed from all patients as per the institutional protocol published earlier [5]. Multiple scrapings were collected from each patient for microscopy and culture. Numbers of scrapings collected for direct microscopic examination varied from 1-3. Whenever three scrapings were taken, they tended to be sequentially collected and respectively stained by 10% potassium hydroxide with 0.1% calcofluor white (KOH+CFW, fluorescence microscopy), Gram and Giemsa stains. The criteria to determine significance of a culture included (i) confluent growth in any solid media; and/or (ii) growth in more than one medium; and/or (iii) growth in one medium with presence of the organism in direct microscopy; and/or (iv) repeat isolation of the organism. For patients undergoing keratoplasty, the corneal tissue removed at keratoplasty was bisected across the ulcer and half of it was submitted to microbiology laboratory in a sterile container. The tissue was minced aseptically using sterile blade and the fragments were inoculated on sheep blood/chocolate agar, brain heart infusion broth, thioglycollate broth and Sabouraud dextrose agar with chloramphenicol. The media were incubated and interpreted as for corneal scrapings [5].

Antifungal topical therapy with 5% natamycin was started for all cases immediately on receiving a positive report of fungal filaments by microscopic examination of the corneal scraping. One hourly topical drops were applied for first three days round the clock followed by two hourly drops during waking hours until resolution of the ulcers. Patients also received 1% atropine sulphate drops. During the study period, under a randomized control study, 6/264 (2.2%) patients had been treated with 1.25% povidone iodine in the same dosage. Systemic ketoconazole (200 mg twice daily) or itraconazole (100 mg twice daily) or fluconazole (150 mg once a day) was prescribed to 158 (58.3%) patients with corneal stromal infiltrate extending beyond one third of the cornea. Additional procedures at the discretion of the clinicians were undertaken for patients not responding to medical therapy and they included therapeutic penetrating keratoplasty (PK), evisceration, and cyanoacrylate glue application with bandage contact lens or anterior chamber wash with amphotericin B.

Post-treatment, an ulcer was considered healed when the epithelial defect was found to be < 1 mm in maximum diameter with slit lamp biomicroscopy and a visible scar. A healing time of < 3 weeks from presentation was considered good result and healing time more than three weeks was considered a poor response.

## Results

**Figure 1:**
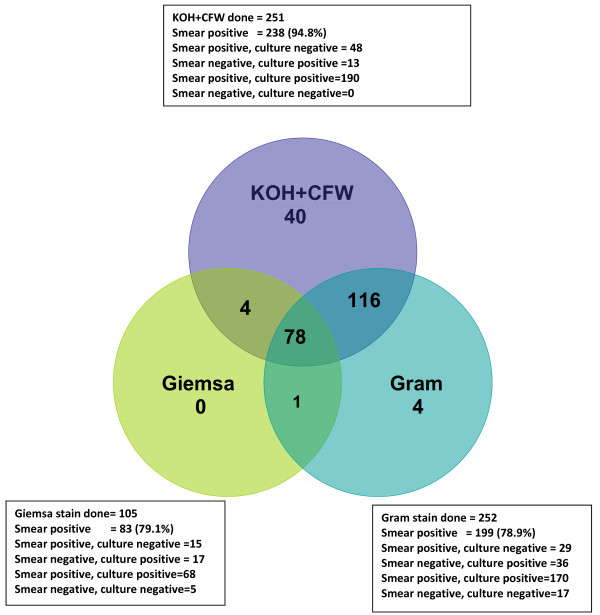
**Venn diagram showing the results of microscopic examination of corneal scrapings from patients with fungal keratitis**. The corneal scrapings were examined by KOH+CFW in 251, by Gram stain in 252 and by Giemsa stain in 105. The figure shows the positivity for fungal filaments by each staining procedure and its correlation to culture results is given in the boxes.

During the study period 997 patients were clinically diagnosed as microbial keratitis and were investigated for bacteria, fungi or parasites. While no organisms were found in 253 (25.3%) clinical samples, 233 (23.4%) were bacterial, 264 (26.4%) were fungal with or without bacteria, 10 (1%) were *Acanthamoeba *and 221 (22.16%) were microsporidia. Sixteen patients (1.6%) had parasitic infection mixed with bacteria (*Acanthamoeba *+ bacteria-4, Microsporidia + bacteria -12). Of the 264 patients with fungal keratitis the diagnosis was made by examination of corneal scrapings in 221 (83.71%), corneal tissue in 7 (2.65%) and both corneal scraping and corneal tissue in 36 (13.6%) patients. Two hundred fifteen of 264 (81.4%) samples grew fungus in culture while 49 corneal scrapings were positive for fungal elements only in direct microscopy and culture were negative. Among the 215 culture positive cases, the growth in culture was considered significant based on confluent growth in 19 cases and based on growth in two or more media in two cases. In 194 cases the smear was positive and culture showed growth in one or more media. In no case repeat culture was required for determining significance of the culture. Clinical diagnosis in the 264 mycotic keratitis varied from fungal in 186 (70.45%), bacterial in 25(9.4%), viral in 4 (1.5%), *Acanthamoeba *in 1 (0.4%) and indeterminate microbial keratitis in 48 (18.2%). Figure 1 shows the results of three methods used for direct microscopy of corneal scrapings. While 251 corneal scrapings were examined by KOH+CFW, 252 had been examined by Gram stain and 105 had been examined by Giemsa stain. Detection of fungal elements in corneal scrapings was 94.8% by KOH+CFW stain. In culture, *Aspergillus *species (27.9%) and *Fusarium *species (23.2%) were the major isolates (Table 1). Of the 215 fungal isolates 172 were from corneal scrapings, 7 were from corneal tissue and 36 were from both corneal scraping and corneal tissue. Age and gender distribution of the patients is shown in Table 2, which shows higher prevalence of mycotic keratitis in males 185 (70%). Concomitant bacterial infection was seen in 45 (17.1%) cases of mycotic keratitis and *Staphylococcus *species (14, 31.1%) was the predominant bacterial pathogen (Table 3). The data pertaining to predisposing factor was not available in 149 (56.4%) patients. Seventy-five (28.4%) patients were farmers. Corneal trauma in 106 (40.15%) patients was identified as the predominant predisposing factor while 6 (2.2%) patients had diabetes and 3 (1.1%) had both.

**Table 1:** Distribution of various fungal species in patients with mycotic keratitis (n = 215)

Type of fungus	Fungal isolates	Number	Percentage (%)
Hyaline fungi	*A. flavus*	34	15.8
	
	*A. fumigatus*	14	6.5
	
	Other *Aspergillus *spp.	12	5.8
	
	*F. solani*	23	10.7
	
	Other *Fusarium *spp.	27	12.6
	
	*Acremonium *spp.	10	4.7
	
	*Colletotrichum *spp.	3	1.4
	
	*Trichosporon *spp.	2	0.9
	
	*Penicillium *spp.	2	0.9
	
	*Paecilomyces *sp.	1	0.5
	
	*Scedosporium apiospermum*	6	2.7
	
	*Aureobasidium pullulans*	1	0.5
	
	*Sepedonium *sp.	1	0.1
	
	*Phialophora verrucosa*	1	0.5
	
	Unidentified hyaline fungus	20	9.3

Dematiaceous fungi	*Curvularia lunata*	6	2.7
	
	Other *Curvularia *spp.	2	0.9
	
	*Cladosporium *spp.	3	1.4
	
	*Lasiodiplodia theobromae*	2	0.9
	
	*Nigrospora *spp.	2	0.9
	
	*Bipolaris spicifera*	2	0.9
	
	*Exophiala *spp.	2	0.9
	
	*Alternaria alternata*	1	0.5
	
	*Cladophialophora *sp.	1	0.5
	
	Unidentified dematiaceous fungus	34	15.8

Yeast	*Candida *spp.	2	0.9
	
	*Rhodotorula glutinis*	1	0.5

**Table 2:** Age and gender distribution of patients with mycotic keratitis (n = 264)

Age in years	Male No. (%)	Female No. (%)	Total No. (%)
0-9	3(1.6)	0	3(1.1)

10-19	4(1.2)	4(5)	8(3)

20-29	23(12.4)	2(2.5)	25(9.5)

30-39	33(17.8)	11(13.9)	44(16.7)

40-49	41(22.2)	18(22.7)	59(22.3)

50-59	38(20.5)	24(30.4)	62(23.5)

60-69	27(14.6)	11(13.9)	38(14.4)

70-79	14(7.6)	6(7.6)	20(7.6)

80 and above	2(1.1)	3(3.8)	5(1.9)

Total	185(70.1)	79(29.9)	264(100)

**Table 3:** Types of bacteria isolated along with fungi in mixed fungal infections (n = 45)

**Sl No**.	Bacterial isolates	Number	Percentage (%)
1	*Staphylococcus *species	14	31.1

2	*Staphylococcus aureus*	3	6.6

3	*Corynebacterium *species	6	12.7

4	*Pseudomonas aeruginosa*	3	6.6

5	*Pseudomonas *species	2	4.4

6	*Pseudomonas *species + *Staphylococcus aureus*	3	6.6

7	*Pseudomonas aeruginosa *+*Micrococcus *species	1	2.2

8	*Corynebacterium *species +*Streptococcus pneumoniae*	2	4.4

9	*Corynebacterium *species +*Klebsiella *species	1	2.2

10	*Staphylococcus aureus *+*Corynebacterium *species	2	4.4

11	*Streptococcus pneumoniae*	4	8.5

12	*Micrococcus *species	2	4.4

13	*Acinetobacter *species	1	2.2

14	*Nocardia asteroides*	1	2.2

Treatment outcome of the patients is shown in Table 4. While 43 (16.28%) patients were lost to follow up (before 4 weeks), the clinical outcome of healed scar was achieved in 94 (35.6%) cases. Twenty nine out of 94 patients (30.9%) had healed scars in < 3 weeks from the date of presentation. Fifty two patients (19.7%) required therapeutic PK, 50 patients required tissue adhesive with bandage contact lens and 9 (3.4%) went for evisceration. In 16 patients there was no change in ulcer at the time of data collection. The mean follow up of the patients was 43 ± 115 days. Analysis of treatment outcome in patients seen before 10 days (early) and after 10 days (late) of presentation showed a significantly (p < 0.001) higher surgical intervention (PK and tissue adhesive) in late cases (Table 4).

**Table 4:** Treatment outcome of patients with mycotic keratitis (n = 264)

Treatment outcome (No. of cases)	Duration of presentation			Duration not known No. (%)
		
	Early (≤ 10 days) No. (%)	Late (> 10 days) No. (%)	P value	
Healed (n = 94)	45 (47.8)	43 (45.6)	0.88	6 (6.5)

Penetrating Keratoplasty (n = 52)	6 (12.9)	40 (76.9)	< 0.001	6 (11.1)

Evisceration (n = 9)	-	5 (55.5)		4 (44.4)

Tissue Adhesive + Bandage Contact Lens (n = 50)	12 (24)	30(60)	< 0.001	8(16)

Status quo (n = 16)	4 (25)	10 (62.5)	0.074	2 (12.5)

Lost to follow up- 43 (16.3%)

## Discussion

This study presents a thorough laboratory and clinical data of a large number of mycotic keratitis patients from eastern India. A comparison of the results with recent data from other parts of the country is shown in Table 5. Fairly large numbers of patients have been analyzed in all of these hospital based published reports. While the prevalence recorded from the southern, western and north-eastern India is between 21-37%, it is only 7.3% from Chandigarh. In a larger study of 3528 microbial keratitis cases in Delhi (north India), the prevalence of fungal keratitis was reported to be 24.3% [9]. Across the country, mycotic keratitis seems to be prevalent in males, in farmers and the most common predisposing factor remains trauma to the cornea. The predominant age is young adults in most studies [2,6] however, some studies [4,7] including the present one find higher prevalence in males between 50-60 years of age.

**Table 5:** Comparison of microbiological and clinical data on fungal keratitis from studies from various parts of India

Parameters	**Bharathi et al (South)**[2]	**Despande et al(West) **[6]	Saha et al **(North East)**[7]	Chander & Sharma **(North)**[4]	Present study (East)
**General**					

Type of study	Retrospective	Prospective	Retrospective	Prospective	Retrospective

Period of study	Sep1999-Aug2002	1988-1996	2008	Jan1987-Dec1992	July2006-Dec2009

Duration	3 years	9 years	1 year	6 years	3.5 years

No. of patients with microbial keratitis	3183	1010	289	730	997

**Microbiology**					

Culture positive for fungus	1171(36.7%)	367(36.3%)	74(25.6%)	53(7.3%)	215(21.5%)

**Nature of sample from which fungus isolated**					

Corneal scraping	1171/1171(100%)	367/367(100%)	41/74(55.4%)	NM	172/215(80.0%)

Corneal tissue	NM	NM	16/74(21.6%)	NM	7/215(3.2%)

Corneal scraping and tissue	NM	NM	NM	NM	36/215(16.7%)

**Analysis of wet mount and different staining methods**					

KOH/CFW positive	1181/1181 (100%)	367(36.3%)	110(38.06%)	NM	238/251(94.8%)

Gram positive	1039/1181 (87.9%)	347(34.4%)	NM	NM	199/252(78.9%)

Giemsa Positive	NM	NM	NM	NM	83/105(79.0%)

Smear positive Culture negative	11/1181(0.9%)	0	36/289(12.4%)	NM	49/257(19.0%)

Fungal culture Positive	1095/1181(92.7)%	36.3%	74/110(67.2%)	7.3%	215/264(81.4%)

Most common Fungal isolate	*Fusarium *spp. (43%)	*Aspergillus* spp. (67.8%)	*Aspergillus* spp. (55%)	*Aspergillus* spp. (40%)	*Aspergillus* spp. (28%)

2^nd ^common isolate	*Aspergillus* spp. (26%)	*Candida *spp. (9.8%)	*Candida *spp. (19%)	*Fusarium *spp. (16.4%)	*Fusarium *spp. (23%)

**Concomitant infection**					

Mixed with bacteria	76/1181(6.4%)	40/367(10.8%)	NM	NM	17%

Most common Bacterial isolate	NM	*Pseudomonas aeruginosa *(66%)	NM	NM	*Staphylococcus* spp. (31%)

**Clinical aspect**					

Age Range	31-40(24%)	31-40	50-60	51-60	50-60 (63%)

Gender	Male (65%)	Male	Male	Male	Male (70%)

Residence Rural	80.3%	NM	NM	NM	79.5%

Urban	20%				21%

Most common predisposing factor	Trauma (92%)	Trauma (55%)	Trauma (48%)	NM	Trauma (40%)

Occupation	Farmer (65%)	NM	NM	NM	Farmer (28%)

**Clinical Diagnosis**					

Fungal keratitis	94.1%	100%	NM	NM	71%

Bacterial keratitis	NM	NM	NM	NM	9%

Viral keratitis	NM	NM	NM	NM	1.5%

*Acanthoemba *keratitis	NM	NM	NM	NM	0.4%

**Treatment given**					

Topical natamycin or voriconazole	NM	NM	100%	NM	94.3%

Systemic ketoconazole	NM	NM	100%	NM	60%

Therapeutic PK	NM	NM	60%	NM	21%

Evisceration	NM	NM	NM	NM	3.4%

**Treatment outcome**					

Scar	NM	NM	40%	NM	35%

Healing time < 3 weeks	NM	NM	NM	NM	32%

Duration of symptoms Early onset < 10 days	NM	NM	NM	NM	92(34.8%)

Late onset > 10 days	NM	NM	NM	NM	172(65.2)

In the hands of experienced cornea specialists the clinical acumen to make a diagnosis of mycotic keratitis varies in different studies from 71-100% [2]. Nevertheless, in all studies, the diagnosis of fungal keratitis is remarkably efficient using relatively simple methods such as potassium hydroxide wet mount and Gram stain. In this study, at 94.8%, the detection of fungal elements in corneal scrapings was very high by microscopy using KOH+CFW stain. Being a retrospective study we are aware that this favorable result could be biased as the first scraping was invariably taken for KOH+CFW stain, especially in cases where clinical suspicion of fungal keratitis was high. However, as supported by several studies [10,11] calcofluor white is indeed a highly reliable and sensitive stain for fungal detection under fluorescence microscope. Since, clinical acumen would vary according to the level of training and experience, it seems appropriate for all practitioners to have the minimum laboratory facility available in their clinic for the management of microbial keratitis. When attempted, it is fairly easy to grow and identify fungi from corneal scrapings and the most common fungi isolated are either *Fusarium *or *Aspergillus *spp [2,4,6,7]. The source of these fungi is obviously the environment which is rife with similar species of fungi [12]. *Candida *spp. are uncommon causes of mycotic keratitis in almost all studies including the present study, though Saha et al have recorded a prevalence of 19% [7]. Only a community based study could show the true prevalence of fungal keratitis. Under the pyramidal model of eye care, currently, L V Prasad Eye Institute is committed to support laboratory facilities in all its secondary centers and provide the minimum requirement of a microscope with potassium hydroxide and Gram stain to examine corneal scrapings from all patients with microbial keratitis. A similar approach at a large scale is recommended.

To determine type of fungi one would require culture facilities in the laboratory. In addition, culture of the corneal scrapings or corneal tissue is the only way to determine mixed fungal and bacterial infections which require combined treatment with antifungal and antibacterial antibiotics. The prevalence of mixed infection varies from 6-10% [2,6] however, this study found 17% patients with mixed infection. While Deshpande et al [6] found *Pseudomonas aeruginosa *as the most common bacteria in mixed infections the commonest organism in this study was *Staphylococcus *spp. Presence of *Pseudomonas *spp. is of particular significance especially in face of the finding of contaminated natamycin eye drops [13]. Treating ophthalmologists would be well advised to take a repeat corneal scraping for culture from a fungal ulcer not responding to treatment, to rule out contamination with *Pseudomonas *spp.

Treatment outcome in mycotic keratitis remains less than satisfactory in most reports [5,7] and this study is no exception. Fifty two patients (19.7%) required therapeutic PK and 9 (3.4%) went for evisceration. Saha *et al *reported PK in 60% of their patients. A large number of patients require therapeutic keratoplasty (PK) despite full treatment with natamycin. Expectedly, early treatment results in favorable outcome. This was obvious in this study as larger number (Table 4) of patients with poor outcome had presented later than 10 days of starting of symptoms. PK and tissue adhesive for impending perforation were seen in significantly more number of patients who presented late. Newer antifungals with greater penetration compared to natamycin have shown promising results in the treatment of mycotic keratitis [14].

## Conclusions

This study highlights that the relative prevalence of mycotic keratitis is less compared to other parts of India and is higher than northern India. The predominant genera of fungi involved (*Aspergillus *and *Fusarium*) are similar across India. Unlike other studies, the prevalence is more in older age groups in this study. The study also shows that fungal keratitis can be easily diagnosed clinically and by laboratory methods, however it remains a therapeutic challenge to the ophthalmologists.

## Competing interests

The authors declare that they have no competing interests.

## Authors' contributions

SKS and SD clinically diagnosed the cases in the out-patient service and collected corneal scrapings for staining and culture. BD, SS and SK helped in examining the slides as well identification of the fungal isolates. They also did retrospective analysis of the data as well as statistical analysis. The manuscript was written by BD and SS and all authors read and approved the final manuscript.

## Pre-publication history

The pre-publication history for this paper can be accessed here:

http://www.biomedcentral.com/1471-2415/11/39/prepub
